# Hhex inhibits cell migration via regulating RHOA/CDC42-CFL1 axis in human lung cancer cells

**DOI:** 10.1186/s12964-021-00763-6

**Published:** 2021-07-28

**Authors:** Xiaopeng Li, Guilin Ma, Wenjie Guo, Ning Mu, Yingying Wang, Xiangguo Liu, Ling Su

**Affiliations:** 1 Shandong Provincial Key Laboratory of Animal Cell and Developmental Biology, School of Life Sciences, Shandong University, Room N8-110, 72 Binhai Road, Qingdao, 266237 China; 2 Shandong Provincial Collaborative Innovation Center of Cell Biology, School of Life Sciences, Shandong Normal University, Jinan, China

**Keywords:** Hhex, RHOA, CDC42, RHOGDIA, p-CFL1

## Abstract

**Background:**

Hhex(human hematopoietically expressed homeobox), also known as PRH, is originally considered as a transcription factor to regulate gene expression due to its homebox domain. Increasing studies show that Hhex plays a significant role in development, including anterior–posterior axis formation, vascular development and HSCs self-renewal etc. Hhex is linked to many diseases such as cancers, leukemia, and type-2 diabetes. Although Hhex is reported to inhibit cell migration and invasion of breast and prostate epithelial cells by upregulating Endoglin expression, the effect and molecular mechanism for lung cancer cell motility regulation remains elusive.

**Methods:**

Human non-small cell lung cancer cells and HEK293FT cells were used to investigate the molecular mechanism of Hhex regulating lung cancer cell migration by using Western blot, immunoprecipitation, wound-healing scratch assay, laser confocal.

**Results:**

Our data indicated that Hhex could inhibit cell migration and cell protrusion formation in lung cancer cells. In addition, Hhex inhibited CFL1 phosphorylation to keep its F-actin-severing activity. RHOGDIA was involved in Hhex-induced CFL1 phosphorylation regulation. Hhex enhanced RHOGDIA interaction with RHOA/CDC42, thus maintaining RHOA/CDC42 at an inactive form.

**Conclusion:**

Collectively, these data indicate that Hhex inhibited the activation of RHOA/CDC42 by enhancing interaction of RHOGDIA with RHOA/CDC42, and then RHOA/ CDC42-p-CFL1 signaling pathway was blocked. Consequently, the formation of Filopodium and Lamellipodium on the cell surface was suppressed, and thus the ability of lung cancer cells to migrate was decreased accordingly. Our findings show Hhex plays an important role in regulating migration of lung cancer cells and may provide a potential target for lung cancer therapy.

**Video abstract**

**Supplementary Information:**

The online version contains supplementary material available at 10.1186/s12964-021-00763-6.

## Background

Lung cancer is the most deadly and second most common cancer in the United States [1]. In worldwide, the incidence and mortality of lung cancer increases fast [2]. The problem with cancer treatment lied in its metastasis, which accounts for 90% of cancer-related deaths [3, 4]. Tumor metastasis from primary sites to secondary sites is a complex process. During cancer metastasis, capacity enhancement of cell motility is an obvious characteristic of tumor cells [5, 6].

Cell movements are associated with RHO-GTPase family, which consists of RAC1, RHOA and CDC42 etc. [7]. Regulated by ARHGDI, RHOGAP and RHOGEF, those small-GTPases are transformed between two different forms: GTP-binding (active) or GDP-binding (inactive) [8]. They regulate cytoskeleton rearrangement, thus enhancing cell migration and invasion. RHOA signaling cascade plays an important role in inducing actin stress fiber and focal adhesion formation [9]. On one hand, RHOA phosphorylate downstream kinase LIMK via Rho-associated kinase (ROCK), and then leads to the phosphorylation of the Cofilin, which is conducive to the aggregation of microfilaments [10]. In addition, RHOA promotes the aggregation of microfilaments by activating PI(4)P5K [11]. RAC1 has been shown to be located in the front of mobile cells and promotes the formation of lamellipodium [12]. The role of CDC42 in cell is mainly to form filopodia. CDC42 is involved in the process of filopodia nucleation, which functions mainly in two aspects: Binding of CDC42 to the WASP complex promotes the activation of ARP2/3 [13, 14]; CDC42 induces nucleation directly via mDia [15]. Overall, RAC1 and CDC42 contribute to lamellipodium and filopodium formation respectively at the leading edge of moving cell [16]. However, RHOA controls stress fibers establishment [17]. Therefore, RHO-GTPases play a crucial role in cell motility regulation.

Cofilin (CFL1), an actin-binding protein, could sever and disassemble actin filaments to reduce cell motility [18]. Its actin-severing activity is suppressed by ser3 phosphorylation. And RHOA/RAC1/CDC42 stimulate downstream effectors to upregulate ser3 phosphorylation of CFL1 and help actin rearrangement [19, 20]. Hence, RHOA/CDC42/RAC1-CFL1 signaling axis affects cell movement via regulating actin polymerization and depolymerization of F-action.

Hhex (human hematopoietically expressed homeobox), also known as PRH, is a 270aa protein which has multiple functions such as regulating gene expression. For example, Hhex can act as a transcription factor which binds to special DNA sequence directly due to its 60aa homebox domain or interacts with other transcription regulators to activate gene expression (or example ENG) or repress gene transcription (for example VEGF, GSC, ESM1 etc.) [21]. Moreover, Hhex can interact with eIF4E to affect translation process [22]. Hhex is also involved in development of embryo like anterior–posterior axis formation and vascular generation [23, 24]. In addition, Hhex is also important for hematopoietic cell differentiation [25, 26] which is related to several diseases such as type 2 diabetes [27] and leukemia [22, 28–30]. Loss of Hhex increases risks of breast cancer, prostate cancer and thyroid cancer [31–34].

In the present study, we demonstrate that Hhex inhibits cell migration and invasion by repressing actin rearrangement via promoting interaction of ARHGDIA with RHOA and CDC42, thus inhibiting RHOA/CDC42-mediated CFL1 phosphorylation. Our findings might highlight the novel role of Hhex in cancer cell migration.

## Material and methods

### Bioinformation analysis

The expression profiles of Hhex in different cancer and normal were analyzed by using TCGA database (http://cancergenome.nih.gov/) and GEPIA online tool (http://gepia.cancer-pku.cn/). Four Oncomine datasets, namely Hou Lung, Landi Lung, Okayama Lung and Selamat Lung were downloaded from Oncomine (https://www.oncomine.org/). The overall survival rate of lung cancer was obtained from the KM plotter website.

### Cell culture

The Calu-1, A549, H1299, H1792 cell lines were obtained from the ATCC (American Type Culture Collection). All four cell lines were grown at 37 °C in a humidified incubator with 5% CO_2_ and maintained in RPMI 1640 (Sigma-Aldrich, R6504). The A549 and Calu-1 cell lines were cultured with 5% NBCS (newborn calf serum) (purchased from Gibco, 1225590). The H299 and H1792 cell lines were cultured with 5% fetal bovine serum (FBS) (Gibco,).

### Antibodies and reagents

Following primary antibodies targeting special proteins were used: Hhex(Thermo Fisher 29154); RHOGDIA (Santa cruz, SC-360); RHOA (CST, #2117P); CDC42 (CST, #2466P); p-CFL1 (Sigma-Aldrich, SAB4300115); HA (Sangon Biotech, AB10004); FLAG (Sigma-Aldrich, F7425); MYC (Sigma-Aldrich,C3956); ACTB (Sigma-Aldrich,A5441); GAPDH (Sigma-Aldrich,G8795). TRITC-phalloidin (Sigma-Aldrich P1951) was used for F-actin staining; and the Transcriptor First Strand cDNA Synthesis Kit (Roche, 04897030001) was used for plasmids construction. The LipoMaxTM Reagent (P/N 32012) and the Polyplus Transfection Reagent (#114-15/1.5 mL) was used for transfection of plasmids and siRNA respectively. Rho Activation Assay Biochem KitTM (cytoskeleton, #BK036) and CDC42 Activation Assay Biochem Kit TM (cytoskeleton, #BK034) was used to detect active RHOA and CDC42 level respectively.

PHEMO buffer (0.025 M HEPES, 0.068 M PIPES, 0.003 M MgCl2·6H2O, 0.015 M EGTA·Na2, 10% DMSO, pH adjusted to 6.8. Additional reagents were added before use, with a final concentration as follows: 0.05% glutaraldehyde, 0.5% Triton X-100, 3.7% formaldehyde) was prepared for fixing cells.

### Plasmids

The primer sequences designed as follows:

HHEX-F: 5′-CGGATCCGCCGCCACCATGCAGTACCCGCACCCC-3′

HHEX-R: 5′-GCTCGAGTCATCCAGCATTAAAATAGCTTT-3′

HHEX-HA-R: 5′-GCTCGAGTCAGGCATAATCGGGTACATCGTAAGGGTATTCCATTCCAGCATTAAAATAGCTTT-3′

RHOGDIA-F: 5′-GGAATTCGCCGCCACCATGGCTGAGCAGGAGCCCAC-3′

RHOGDIA-FLAG-R:

5′-GCTCGAGTCACTTGTCGTCATCGTCTTTGTAGTCGTCCTTCCAGTCCTTCTTG-3′

These genes were cloned by PCR using cDNA as template, then inserted into pcDNA3.1 vector.

### siRNA

The HHEX siRNAs were synthesized by GenePharma (Shanghai, China) which targeted sequences:

#1: 5′-GCCCAGUGAACAGAAUAAA-3′

#2: 5′-GGUGCUUCUUUGGAUAGCUUU-3′

### Western blot analysis

Western blot analysis was performed as described previously [35]. The primary antibodies used were described as above.

### Wound-healing scratch assay

Cells were cultured in a 6-well plate then transfected with plasmids or siRNA. When cells reached monolayer confluency, all cells were treated with proliferation inhibitors *mitomycin*-C (10 μg/ml)1 h prior to performing the scratch assay, and then a 200uL pipette tip was used to scratch across the bottom of plate. To remove these suspended cells, all wells were washed three times by PBS. After that, cells were maintained with medium described above including 1% FBS. Then, the cells were cultured for 48–72 h at 37 °C in a humidified incubator with 5% CO_2_. A microscope with a digital camera were needed to photograph the sites scratched. Images were obtained at the indicated time points and migration rate was calculated.

### Transwell assay

Transwell assays were conducted using 24-well transwell chamber coated. The 10% FBS medium was added in the lower chamber. Then, the siRNA or siCTRL mixture was pipetted to serum-free medium containing 4*10^4^ cells and then transferred to the upper chamber. After incubation at 37 ℃ with 5% CO2 for 12 h, the cells on the lower surface were fixed with 4% paraformaldehyde for 0.5 h and stained with 0.1% crystal violet for 0.5 h. The migrated cells were counted in three random fields under a light microscope and quantified using the ImageJ software, then normalized against the NC treated cells to determine the relative ratio.

### RHO/CDC42 activation assay

GTP–RhoA/CDC42 pulldown assays were performed with RhoA/CDC42 Pulldown Activation Assay Kit (Cytoskeleton) according to the manufacturer’s instructions. This assay uses the Rho binding domain (also called the RBD) of the Rho effector protein rhotekin. The RBD protein motif has been shown to bind specifically to the GTP-bound form of Rho. The RBD region of rhotekin has a high affinity for GTP-Rho. The amount of activated Rho is determined by a Western blot using a Rho specific antibody. Cells were cultured in a 10 cm dish then transfected with plasmids or siRNA when cells grown to about 40%. After 8 h, old medium was removed and 1 × PBS was used to wash dish twice. After that, the dish was filled with fresh medium and the cells were cultured for 48-72 h at 37 °C in a humidified incubator with 5% CO2 for 16 h. Then, cells were collected and lysed. lysed and processed for the pulldown assay according to the manufacturer's instructions.

### F-actin staining assay

F-actin staining assay was performed as described previously [36].

### Statistical analysis

GraphPad Prism software was used for statistical analysis. All data were presented as the mean ± SD. Differences between groups were identified using two-sided Student’s t-test. The Kaplan–Meier curves for survival analyses were determined using the log-rank test. *P* < 0.05 was considered statistically significant.

## Results

### Hhex was downregulated and inhibited cell migration in lung cancer cells

**Fig. 1:**
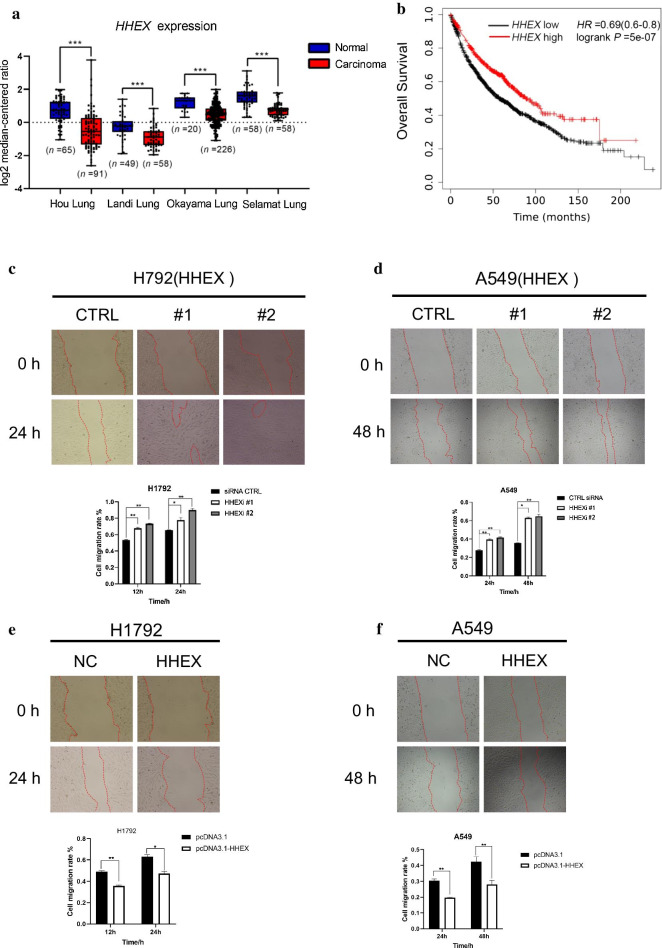
Hhex was downregulated and inhibited cell migration in lung cancer cells. **a** Box plots of Hhex mRNA levels determined from four Oncomine datasets, namely Hou Lung, Landi Lung, Okayama Lung and Selamat Lung, (***P < 0.001; *P* values were obtained using two-tailed Student’s t-tests). **b** KM plotter analysis of the relation between Hhex gene expression and (OS) in lung cancer patients. **c** Control siRNA (CTRL) or HHEX siRNA (#1) and (#2) was transfected into H1792 cells. When cells reached monolayer confluency, all cells were treated with proliferation inhibitors mitomycin-C (10 μg/ml)1 h prior to performing the scratch assay. And the images of these scratched sites were obtained every 12 h to recorded width changes. The picture showed above represented time points at 0 h and 24 h for each group. Western blotting of 24 h whole-cell extracts was performed. And t-test was used to analyze the differences between the treatment groups. Data are presented as means ± S.D. *P < 0.05; **P < 0.01 (n = 5) (**d**) A549 cells were treated similar to H1792 cells. The picture showed above represented time points at 0 h and 48 h for each group. Data are presented as means ± S.D. *P < 0.05; **P < 0.01 (n = 5) (**e**), **f** Hhex was overexpressed in H1792 cells and A549 cells, then wound-healing scratch experiments were performed as same as (**c**, **d**), data are presented as means ± S.D. *P < 0.05; **P < 0.01 (n = 5)

Previous studies have suggested that Hhex regulated cell migration via upregulating ENG expression and downregulating GSC (a critical transcription factor for EMT) expression in breast cancer cells [33]. In order to explore the role Hhex plays in lung cancer, the expression of Hhex was analyzed using TCGA database and GEPIA online tool. We found that level of Hhex reduced in multiple cancer tissues was reduced including BLCA, BRCA, COAD, KICH, KIRP, LUAD, LUSC, READ, THCA and UCEC, compared with normal tissues in the GEPIA database (Additional file 1: Figure S1). In addition, four Oncomine datasets, namely Hou Lung, Landi Lung, Okayama Lung and Selamat Lung, were used to examine the mRNA expression of Hhex (Fig. 1a). The data showed that the mRNA levels of Hhex were significantly lower in lung cancer tissues than that in normal tissues. Next, the prognostic significance associated with the expression of Hhex was evaluated using the KM plotter database. The results showed that higher expression of Hhex was correlated with higher overall survival (Fig. 1b).

To investigate whether Hhex can influence cell motility in lung cancer cells, series of wound-healing scratch assays and transwell migration assays were conducted. Hhex knockdown and overexpression were confirmed by Western Blot analysis (Additional file 2: Figure S2). Cell migration was significant enhanced after Hhex knockdown in H1792 cells and A549 cells (Fig. 1c, d; Additional file 3: Figure S3.a, b). Conversely, cell capability of migration was reduced distinctly after overexpression of Hhex in H1792 cells and A549 cells (Fig. 1e, f; Additional file 3: Figure S3.a, b). Our results suggested that Hhex can reduce cell migration in lung cancer cells.

Hhex is regarded as a critical transcription factor. To test whether Hhex inhibits cell migration through the transcriptional properties of Hhex, we generated the plasmid expressing HA-tagged Hhex whose NLS (amino acids 137–197) region was deleted, and the plasmid was designated as pcDNA3.1-HHEX-∆(137–197). The plasmids of pcDNA3.1-HHEX and pcDNA3.1-HHEX-∆(137–197) were transfected into H1792 cells, and the levels of Hhex in the cytoplasm and nucleus was examined using nucleocytoplasmic separation analysis. The results showed that Hhex level in the nucleus was reduced while Hhex level in the cytoplasm was increased in the cells transfected with pcDNA3.1-HHEX-∆(137–197) plasmid (Additional file 4: Figure S4.b). Subsequently, after the plasmids of pcDNA3.1 and pcDNA3.1-HHEX-∆(137–197) were transfected into H1792 and A549 cells for 24 h, cell migration assay was conducted by Transwell method and scratch assay. The data showed cell capability of migration was reduced after overexpression of HHEX-∆(137–197) in H1792 and A549 cells, suggesting Hhex inhibits cell migration independently of its transcriptional properties (Additional file 4: Figure S4.a, c).

### Cell protrusion formation was negatively correlated with Hhex expression

**Fig. 2:**
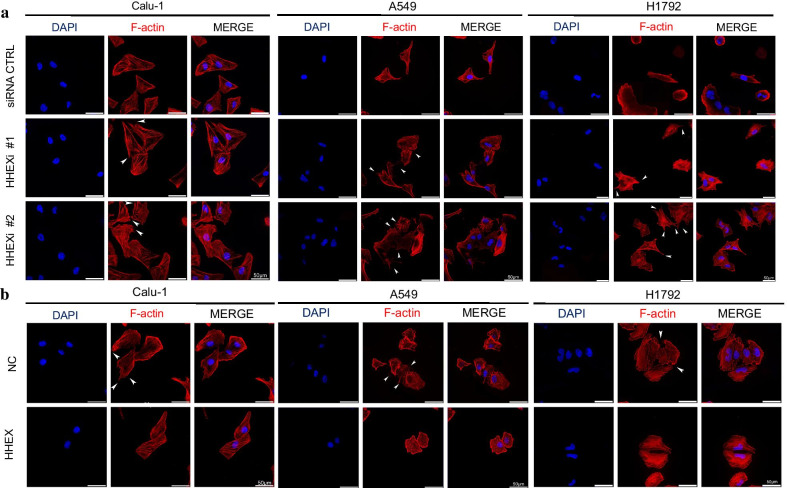
Hhex repressed cell protrusion formation. **a** Calu-1, A549, H1792 cells were transfected with control siRNA or HHEX siRNA. After 24 h, cells were reseeded onto adhesive microscope slides. When cells adhered to slides completely then were fixed with PHEMO buffer. The nuclei were stained with DAPI (blue) and F-actin was stained with TRITC-conjugated phalloidin (red). Scale bars, 50 μm. **b** Calu-1, A549, H1792 cells were transfected with pcDNA3.1 or pcDNA3.1-HHEX, and the cells were reseeded onto adhesive microscope slides after 24 h of transfection. F-actin was stained with TRITC-conjugated phalloidin (red), and the nuclei were visualized with DAPI (blue). Arrows stand for the typical protrusions, scale bars, 50 μm

Protrusions were enriched at the edge of migrating cells. Those actin-abundant structures contained at least three subtypes: invadopodia, filopodia and lamellipodium. Filopodia and lamellipodium were common in two-dimension environments, which generated force to drive cell to move. To test whether Hhex had relationship with protrusions formation, microfilaments of treated cells were stained by TRITC-phalloidin and then visualized with a laser scanning confocal microscope to show changes of cell morphology. The images showed increasing protrusions were exhibited in Hhex siRNA transfected Calu-1, A549 and H1792 cells (Fig. 2a). However, more smooth edges were found in Hhex overexpressed Calu-1, A549 and H1792 cells (Fig. 2b). These data indicated that Hhex expression was negatively correlated with cell protrusion formation.

### Hhex inhibited RHOA and CDC42 activation

**Fig. 3:**
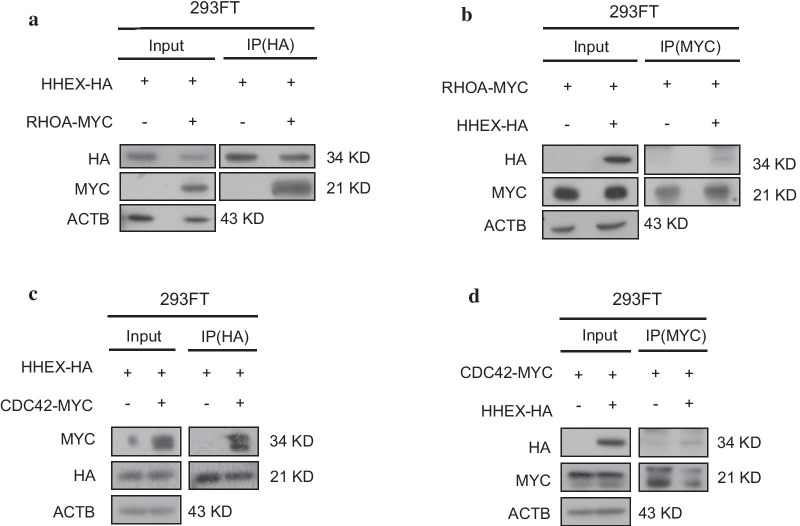
Hhex interacted with RHOA and CDC42. **a** 293FT cells were co-transfected with plasmids of pcDNA3.1 and pcDNA3.1-HHEX-HA or plasmids of pcDNA3.1-HHEX-HA and pcDNA3.1-RHOA-MYC, cell lysate was immunoprecipitated with anti-HA antibody, Hhex and RHOA were detected by western blot. The images are representative of three independent experiments with similar results. **b** 293FT cells were co-transfected with plasmids of pcDNA3.1 and pcDNA3.1-RHOA-MYC or plasmids of pcDNA3.1-HHEX-HA and pcDNA3.1-RHOA-MYC, cell lysate was immunoprecipitated with anti-MYC antibody, Hhex and RHOA were detected by western blot. The images are representative of three independent experiments with similar results. **c** 293FT cells were co-transfected with plasmids of pcDNA3.1 and pcDNA3.1-HHEX-HA or plasmids of pcDNA3.1-HHEX-HA and pcDNA3.1-CDC42-MYC, cell lysate was immunoprecipitated with anti-HA antibody, Hhex and CDC42 were detected by western blot. The images are representative of three independent experiments with similar results. **d** 293FT cells were co-transfected with plasmids of pcDNA3.1 and pcDNA3.1-CDC42-MYC or plasmids of pcDNA3.1-HHEX-HA and pcDNA3.1-RHOA-MYC, cell lysate was immunoprecipitated with anti-MYC antibody, Hhex and CDC42 were detected by western blot. The images are representative of three independent experiments with similar results

**Fig. 4:**
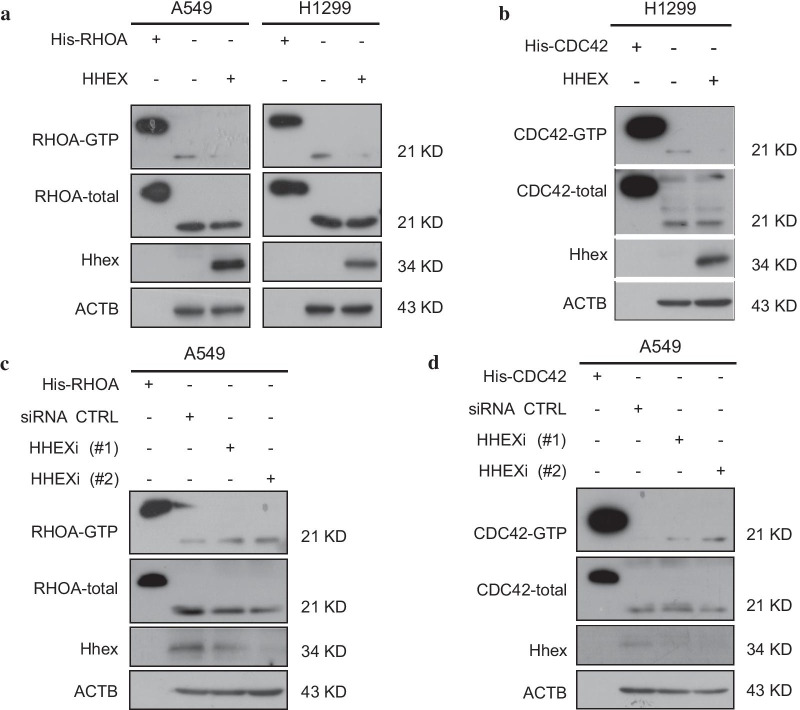
Hhex inhibited RHOA and CDC42 activation. **a** pcDNA3.1 or pcDNA3.1-HHEX plasmid was transfected into A549 cells and H1299 cells for 24 h. RHOA activation assay kit was used for detecting RHOA-GTP active form. The kit supplies His-RHOA protein as a control. **b** H1299 cells were transfected with pcDNA3.1 or pcDNA3.1-HHEX plasmid respectively for 24 h. Then, active CDC42-GTP level was tested by CDC42 activation assay kit. **c** Hhex was knocked down in A549 cells, RHOA-GTP assay was conducted. **d** Hhex was knocked down in A549 cells, CDC42-GTP assay was performed. Results are from one representative experiment of at least three

RHOGTPases regulate protrusion formation. Those small GTPases transform between two distinct forms: GTP-binding(active) or GDP-binding(inactive). Active RHOGTPases transmit upstream signals to remodel microfilaments, promoting pseudopodium formation. To investigate whether Hhex inhibited protrusion formation through repressing RHOGTPases activation, we examined interactions between Hhex and RHOGTPases. Interactions between Hhex and RHOA /CDC42 were confirmed by co-IP assay in 293FT cells (Fig. 3). Moreover, RHOA and CDC42 activation assays were conducted to test Hhex’s role in regulating RHOA and CDC42 activation. RHOA activation was restrained remarkably by Hhex overexpression in A549 and H1299 cell lines (Fig. 4a). The results also suggested CDC42 activation was negatively regulated by Hhex increase in H1299 cells (Fig. 4b). Contrarily, reducing Hhex expression with knockdown method increased active RHOA and active CDC42 level (Fig. 4c, d). Generally, these data indicated Hhex could inhibit RHOA and CDC42 activation in lung cancer cells.

### Hhex reduced CFL1 phosphorylation

**Fig. 5:**
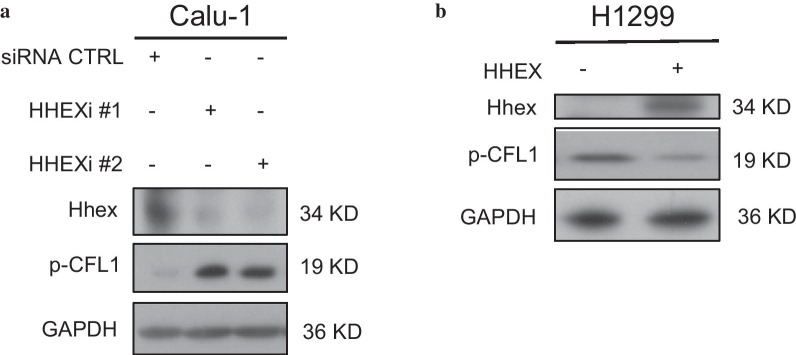
Hhex inhibited CFL1 phosphorylation. **a** Calu-1 cells were transfected with control siRNA or HHEX siRNA for 48 h, and then western blot was performed to detect the level of CFL1 phosphorylation. **b** Overexpression of Hhex by transfection of the pcDNA3.1-HHEX plasmid in H1299 cells. CFL1 phosphorylation was detected by western blot. The images are representative of three independent experiments with similar results

During cell migration, microfilament remodeling was influenced by multiple important factors such as actin-binding proteins, including ARP2/3, Formin, CFL1 etc. For example, CFL1 had actin-severing activity to accelerate net actin depolymerization. Usually, this function was adjusted through pH changes, binding with PI(4,5)P2 and its ser3 phosphorylation. CFL1 phosphorylation at ser3 led to an inactive form [19, 20]. In order to investigate potential effect of CFL1 in Hhex-mediated protrusion repression, Hhex was silenced in Calu-1 and H1299 cells. The Western Blot results showed that p-CFL1 level increased in these Hhex knockdown cells (Fig. 5a, b). Taken together, our works suggested Hhex repressed CFL1 phosphorylation in lung cancer cells.

### Hhex and RHOGDI co-ordinated to inhibit CFL1 phosphorylation

**Fig. 6:**
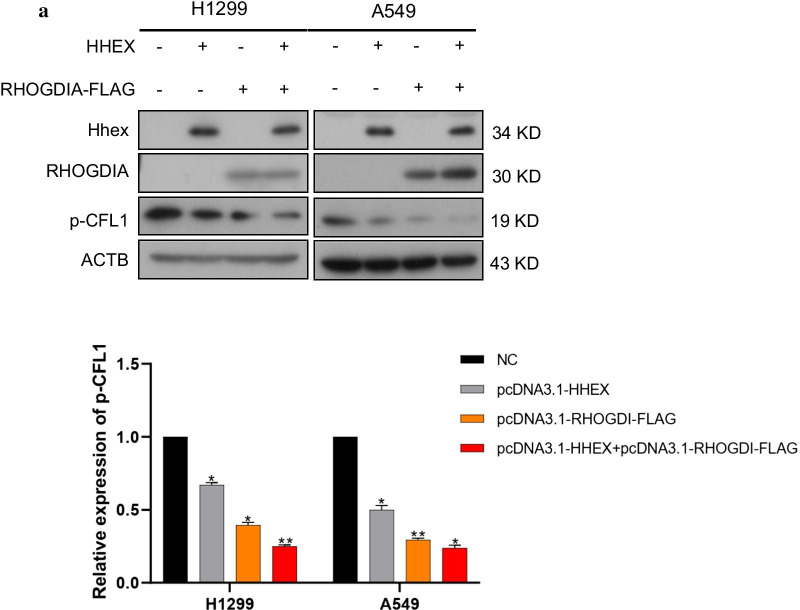
Hhex and RHOGDI co-ordinated to inhibit CFL1 phosphorylation. **a** After co-transfection of Hhex and RHOGDIA in H1299 and A549 cells, CFL1 phosphorylation was detected by western blot. The images are representative of three independent experiments with similar results

Cell motility was associated with RHOA, CDC42, RAC1 and other RHO-GTPases. Nevertheless, the activities of these small GTPase were regulated by series of its binding protein, like RHOGAP, RHOGEF and RHOGDI. RHOGDI was able to keep RHOA/CDC42/RAC1 in inactive form by preventing from GDP disassociation. Hence, RHOGDI could repress RHOA/CDC42/RAC1-CFL1 signaling pathway. To understand whether RHOGDI was required for Hhex-induced p-CFL1 downregulation, RHOGDIA (a universal number of RHOGDI family) and Hhex were overexpressed simultaneously in H1299 cells, the Western Blot results showed that Hhex enhanced RHOGDI-induced p-CEL1 decrease. Consistent results were obtained in A549 cells (Fig. 6). In summary, these data showed that RHOGDI was involved in Hhex-dependent CFL1 phosphorylation regulation. Hhex could accelerated p-CFL1 decrease which was regulated by RHOGDI.

### Hhex enhanced interaction of RHOGDIA with RHOA/CDC42

**Fig. 7:**
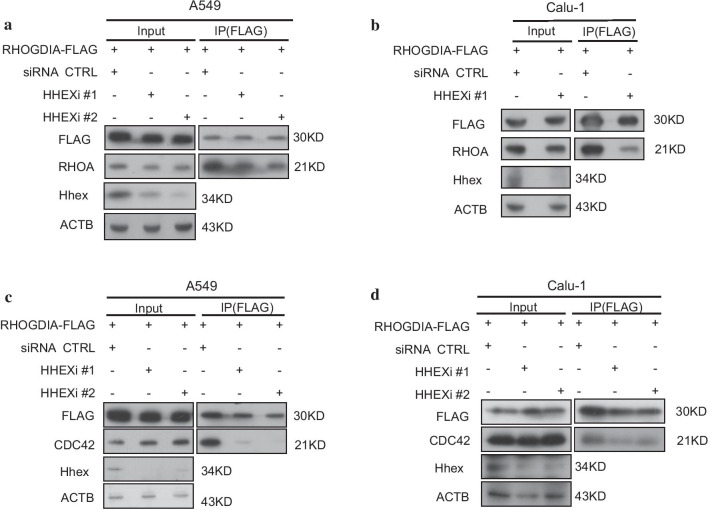
Hhex enhanced interaction of RHOGDIA with RHOA/CDC42. **a** HHEX siRNA-RHOGDIA were co-transfected into A549. Cell lysate was immunoprecipitated with anti-FLAG antibody, Hhex, RHOA were detected by western blot. The images are representative of three independent experiments with similar results. **b** HHEX siRNA and pcDNA3.1-RHOGDIA were co-transfected into Calu-1. Cell lysate was immunoprecipitated with anti-FLAG antibody, Hhex, RHOA were detected by western blot. The images are representative of three independent experiments with similar results. **c** A549 cells were conducted same treatments as (**a**), Hhex and CDC42 were detected by western blot. The images are representative of three independent experiments with similar results. **d** Calu-1 cells were conducted same treatments as (**a**), Hhex and CDC42 were detected by western blot. The images are representative of three independent experiments with similar results

Hhex can repress p-CFL1 level. However, whether Hhex conducted this effect via RHOGDI was still unknown. Co-IP experiment did not show a direct interaction of Hhex with RHOGDI. However, we found that Hhex could bind with RHOA and CDC42. Such interaction may exclude the affinity of RHOGDI with RHO-GTPases. To test this hypothesis, co-IP was conducted and the results showed that RHOA and CDC42 binding to RHOGDI were obviously decreased when Hhex was knocked down in A549 and Calu-1 cells respectively (Fig. 7a–d), indicating that Hhex knockdown repressed interaction of RHOGDI with RHOA/CDC42. Collectively these data demonstrated that Hhex enhanced physical interaction of RHOGDI with RHOA/CDC42, thus keeping RHOA/CDC42 in an inactive form. After RhoGDIA was overexpressed in 293 T cells using transfecting pcDNA3.1-RhoGDIA plasmid, co-IP assay was performed. The results indicted the RHOA and CDC42 binding to Hhex were reduced slightly, indicating that RhoGDIA may inhibit the Hhex-RhoA interaction minimally (Additional file 5: Fig. S5 a, b).

## Discussion

Haematopoietically expressed homeobox (Hhex) is an essential transcription factor in embryonic development and in the adult. Hhex can activate or repress gene expression depending on the target gene. Hhex can also bind to other transcription factors and co-regulate specific target genes either directly through DNA binding, or indirectly through effects on the activity of its partner proteins, e.g., Hhex can repress transcription of target genes via the recruitment of members of the Groucho/TLE family of co-repressor proteins [21]. Hhex can integrate with eIF4E, leading to inhibiting translation of the CyclinD1, which subsequently leads to inhibiting cell cycle [22].

Hhex is known as a protein related to many carcinomas, like breast cancer, thyroid cancer, etc. In these cancers, Hhex regulated growth, migration and invasion of tumor cells. Hhex has been shown to be a transcriptional repressor. For example, motility of breast cancer cells is obviously repressed by Hhex because it can act as a transcription factors to upregulated Endogin expression [33]. In endothelial cells, overexpression of Hhex also controls VEGF-signalling genes and alters cell migration and invasion [37]. However, whether this protein can regulate cell migration of lung cancer cells is still unclear.

In our research, Hhex silencing was used to repress its expression in Calu-1 cells and A549 cells. After Hhex knockdown, migrations of these two cell lines were increased distinctly. Conversely, Hhex overexpression made lung cancer cells lose partial abilities of movement. Moreover, Our results showed that Hhex inhibited the formation of protrusions in NSCLC cells. Given that Hhex acts in the nucleus as a transcription factor, then whether Hhex inhibits cell migration by its transcriptional properties. We generated the plasmid expressing HA-tagged Hhex whose NLS (amino acids 137–197) region was deleted, and the plasmid was designated as pcDNA3.1-HHEX-∆(137–197). The data showed cell capability of migration was reduced after overexpression of HHEX-∆(137–197) in H1792 and A549 cells, suggesting Hhex inhibits cell migration independently of its transcriptional properties.

The migratory machinery of cancer cells is depended on interactions between specific cell cytoskeletal proteins coordinated by small GTPases, such as the Rho family of proteins (RHOA, CDC42 and RAC-1 [38]. CDC42 is usually activated at the leading edge of the pseudopodia to facilitate the formation of protrusions to promote metastasis [39]. RHOA, as a molecular switch in transducing extracellular signals to actin and microtubule cytoskeleton, is an important part of cell migration [40]. Thus, we conjecture whether Hhex inhibits cell migration by RHOA/CDC42. Different from the mechanism of Hhex regulation in breast cancer cells, our results showed that Hhex was associated with RHOA and CDC42 activation. The RHOGTPase activation assay showed RHOA and CDC42 activation were significantly decreased after Hhex overexpression. However, reduced Hhex resulted in a remarkable increase of active RHOA and CDC42 level. These data implied that Hhex inhibited protrusion formation by repressing RHOGTPases activation.

p-CFL1 was an important downstream effector of RHOA and CDC42. This protein was considered to promote actin rearrangement. CFL1 phosphorylation was remarkably increased when Hhex was overexpressed in H1299 cells and A549 cells. As expected, knockdown of Hhex resulted in noteworthy downregulation of p-CFL1 level in Calu-1 cells. Taken together, our data suggested that Hhex inhibited protrusion formation via repressing RHOA/CDC42-p-CFL1 pathway.

We also found that RHOGDIA was involved in cell migration which was regulated by Hhex because the decrease of p-CFL caused by RHOGDIA overexpression were restored by Hhex knockdown in A549 cells and Calu-1 cells. When Hhex and RHOGDIA were co-overexpressed, CFL1 phosphorylation were further reduced. RHOGDIA is an RHO-GTPase binding protein which inhibites GDP dissociation from RHOA/CDC42/RAC1 to keep them in inactive state [41]. Although we didn't detect the direct interaction of Hhex with RHOGDIA, Hhex silencing does reduce the interaction of RHOGDIA with RHOA or CDC42 in an unknown manner. We propose perhaps Hhex regulates another factor to inhibit the RHOA or CDC42 through RHOGDIA. It has been recently demonstrated that Hhex nuclear localization is reduced in cancer cells. GPC3 can binds to CD81, leading to CD81-Hhex binding decreases, resulting in nuclear translocation of Hhex and transcriptional repression [42]. Hhex can also bind to other transcription factors and co-regulate specific target genes either directly through DNA binding, or indirectly through effects on the activity of its partner proteins. Hhex can repress transcription of target genes via the recruitment of members of the Groucho/TLE family of co-repressor proteins [21]. The regulation of Hhex on cell migration may be the common result of the combined action of multiple functions. The roles Hhex plays on earth during lung cancer cell migration need further study.

## Conclusion

In summary, our work uncovered that Hhex negatively regulated cell migration of lung cancer cells by enhancing RHOGDIA interaction with RHOA/CDC42, which reduced downstream effector CF1 phosphorylation, thus inhibiting cell protrusions formation. Given Hhex is associated with lung cancer cell migration, it might be a potential marker for lung cancer diagnosis screening and prognosis evaluation.

## Supplementary Information

**Additional file 1. Figure S1** Hhex was downregulated in lung cancer cells. Box plots of Hhex mRNA levels determined in BLCA, BRCA, COAD, KICH, KIRP, LUAD, LUSC, READ, THCA and UCEC, compared to normal tissues in the GEPIA database (***P < 0.001; *P*-values were obtained using two-tailed Student’s t-tests). BLCA: Bladder Urothelial Carcinoma; BRCA: Breast invasive carcinoma; COAD: Colon adenocarcinoma; KICH: Kidney Chromophobe; KIRP: Kidney renal papillary cell carcinoma; LUAD: Lung adenocarcinoma; LUSC: Lung squamous cell carcinoma; READ: Rectum adenocarcinoma; THCA: Thyroid carcinoma; UCEC: Uterine Corpus Endometrial Carcinoma.

**Additional file 2. Figure S2**. Efficient Hhex knockdown and overexpression were confirmed by Western Blot analysis, related to Figure 1. A549, H1792 cells were transfected with control (CTRL) or HHEX siRNA, pcDNA3.1 or pcDNA3.1-HHEX for 24h and then subjected to western blot analysis. The images are representative of three independent experiments with similar results.

**Additional file 3. Figure S3** Hhex inhibited cell migration in lung cancer cells. (**a**) Hhex knockdown and overexpression was performed in H1792 cells for 24 h and then 4*104 cells were seeded in transwell chamber to conduct migration assay. Cells were then stained using crystal violet after incubated for 12 h. Statistical image shows the number of cells migrated. t-test was used to analyze the differences between the treatment groups. Data are presented as means S.D. *P < 0.05; **P < 0.01 (n=3). (**b**) Hhex knockdown and overexpression was performed in A549 cells for 24 h and then 4*104 cells were seeded in transwell chamber to conduct migration assay. Cells were then stained using crystal violet after incubated for 12 h. Statistical image shows the number of cells migrated. t-test was used to analyze the differences between the treatment groups. Data are presented as means S.D. *P < 0.05; **P < 0.01 (n=3).

**Additional file 4. Figure S4**. Hhex inhibited cell migration independently of its transcriptional properties. (**a**) pcDNA3.1 and pcDNA3.1-HHEX-Δ(137-197)-HA was transfected into H1792 and A549 cells. When cells reached monolayer confluency, all cells were treated with proliferation inhibitors mitomycin-C (10 μg/ml)1 h prior to performing the scratch assay. And the images of these scratched sites were obtained every 12 h to recorded width changes. The picture showed above represented time points at 0 h and 24 h for each group. (**b**) pcDNA3.1-HHEX and pcDNA3.1-HHEX-Δ(137-197) were transfected into H1792 cells, and cytoplasm and nucleus was separated using nucleocytoplasmic separation analysis, and then subjected to western blot analysis. (**c**) pcDNA3.1 and pcDNA3.1-HHEX-Δ(137-197) was transfected into H1792 and A549 cells for 24 h and then 4*104 cells were seeded in transwell chamber to conduct migration assay. Cells were then stained using crystal violet after incubated for 12 h. Statistical image shows the number of cells migrated. t-test was used to analyze the differences between the treatment groups. Data are presented as means S.D. *P < 0.05; **P < 0.01 (n=3).

**Additional file 5. Figure S5** RhoGDIA inhibited the Hhex-RhoA/CDC42 interaction minimally. (**a**) pcDNA3.1-HHEX-HA, pcDNA3.1-RHOGDIA-FLAG and pcDNA3.1-CDC42-MYC were co-transfected into HEK 293T cell. Cell lysate was immunoprecipitated with anti-HA antibody, HA, MYC, ACTB, FLAG were detected by western blot. The images are representative of three independent experiments with similar results. (**b**) pcDNA3.1-HHEX-HA, pcDNA3.1-RHOGDIA-FLAG and pcDNA3.1-RHOA-MYC were co-transfected into HEK 293T cell. Cell lysate was immunoprecipitated with anti-HA antibody, HA, MYC, ACTB, FLAG were detected by western blot. The images are representative of three independent experiments with similar results.

## Data Availability

Data sharing not applicable to this article as no datasets were generated or analysed during the current study.

## Abbreviations

NSCLC: Non-small cell lung cancer cell
Hhex: Human hematopoietically expressed homeobox
CDC42: Cell division cycle 42
RHOA: Ras homolog family member A
RAC1: Rac family small GTPase 1
CFL1: Cofilin
RHOGDI: Rho GDP dissociation inhibitor
ARHGAP1: Rho GTPase activating protein 1
ARHGEF: Rho guanine nucleotide exchange factors
BLCA: Bladder Urothelial Carcinoma
BRCA: Breast invasive carcinoma
COAD: Colon adenocarcinoma
KICH: Kidney Chromophobe
KIRP: Kidney renal papillary cell carcinoma
LUAD: Lung adenocarcinoma
LUSC: Lung squamous cell carcinoma
READ: Rectum adenocarcinoma
THCA: Thyroid carcinoma
UCEC: Uterine Corpus Endometrial Carcinoma
