# Supplementary figures and images for: Hhex inhibits cell migration via regulating RHOA/CDC42-CFL1 axis in human lung cancer cells
Source: Cell Commun Signal. 2021 Jul 28;19:80. doi: 10.1186/s12964-021-00763-6 (PMC8320060; doi:10.1186/s12964-021-00763-6)

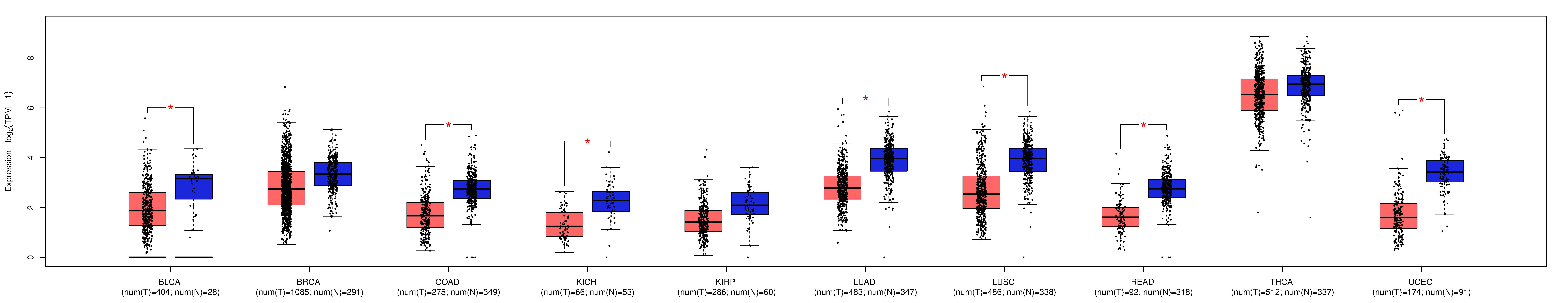

Supplement: Supplementary file 2 — Additional file 1. Figure S1 Hhex was downregulated in lung cancer cells. Box plots of Hhex mRNA levels determined in BLCA, BRCA, COAD, KICH, KIRP, LUAD, LUSC, READ, THCA and UCEC, compared to normal tissues in the GEPIA database (***P < 0.001; P-values were obtained using two-tailed Student’s t-tests). BLCA: Bladder Urothelial Carcinoma; BRCA: Breast invasive carcinoma; COAD: Colon adenocarcinoma; KICH: Kidney Chromophobe; KIRP: Kidney renal papillary cell carcinoma; LUAD: Lung adenocarcinoma; LUSC: Lung squamous cell carcinoma; READ: Rectum adenocarcinoma; THCA: Thyroid carcinoma; UCEC: Uterine Corpus Endometrial Carcinoma. [file 12964_2021_763_MOESM2_ESM.tif]

## Slide 1
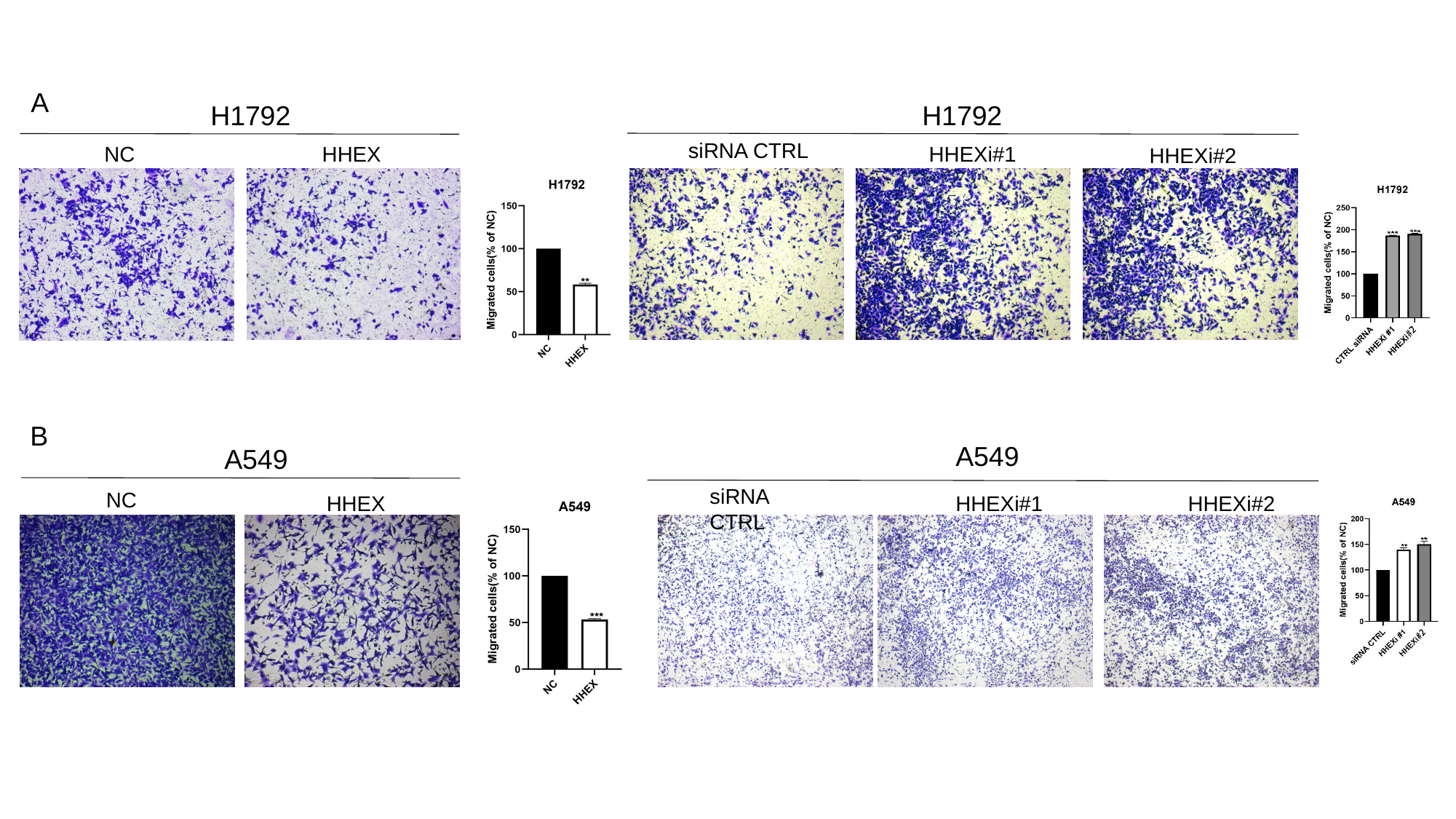

siRNA CTRL
A
H1792
H1792
NC
HHEX
HHEXi#1
HHEXi#2
B
A549
siRNA CTRL
A549
NC
HHEX
HHEXi#1
HHEXi#2

Supplement: Supplementary file 4 — Additional file 3. Figure S3 Hhex inhibited cell migration in lung cancer cells. (a) Hhex knockdown and overexpression was performed in H1792 cells for 24 h and then 4*104 cells were seeded in transwell chamber to conduct migration assay. Cells were then stained using crystal violet after incubated for 12 h. Statistical image shows the number of cells migrated. t-test was used to analyze the differences between the treatment groups. Data are presented as means S.D. *P < 0.05; **P < 0.01 (n=3). (b) Hhex knockdown and overexpression was performed in A549 cells for 24 h and then 4*104 cells were seeded in transwell chamber to conduct migration assay. Cells were then stained using crystal violet after incubated for 12 h. Statistical image shows the number of cells migrated. t-test was used to analyze the differences between the treatment groups. Data are presented as means S.D. *P < 0.05; **P < 0.01 (n=3). [file 12964_2021_763_MOESM4_ESM.pptx]

## Slide 1
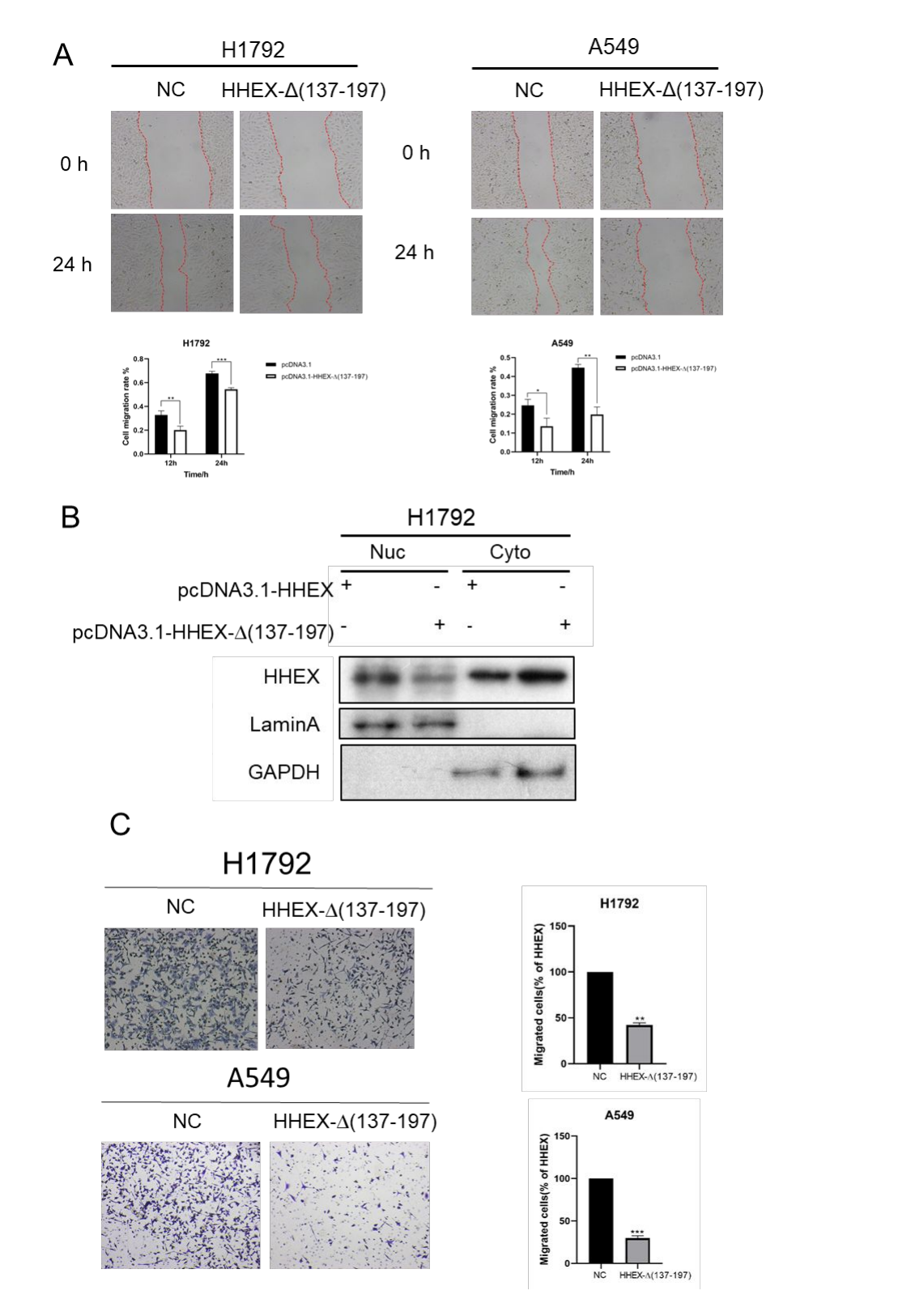

Supplement: Supplementary file 5 — Additional file 4. Figure S4. Hhex inhibited cell migration independently of its transcriptional properties. (a) pcDNA3.1 and pcDNA3.1-HHEX-Δ(137-197)-HA was transfected into H1792 and A549 cells. When cells reached monolayer confluency, all cells were treated with proliferation inhibitors mitomycin-C (10 μg/ml)1 h prior to performing the scratch assay. And the images of these scratched sites were obtained every 12 h to recorded width changes. The picture showed above represented time points at 0 h and 24 h for each group. (b) pcDNA3.1-HHEX and pcDNA3.1-HHEX-Δ(137-197) were transfected into H1792 cells, and cytoplasm and nucleus was separated using nucleocytoplasmic separation analysis, and then subjected to western blot analysis. (c) pcDNA3.1 and pcDNA3.1-HHEX-Δ(137-197) was transfected into H1792 and A549 cells for 24 h and then 4*104 cells were seeded in transwell chamber to conduct migration assay. Cells were then stained using crystal violet after incubated for 12 h. Statistical image shows the number of cells migrated. t-test was used to analyze the differences between the treatment groups. Data are presented as means S.D. *P < 0.05; **P < 0.01 (n=3). [file 12964_2021_763_MOESM5_ESM.pptx]
