# Supplementary material for: Hhex inhibits cell migration via regulating RHOA/CDC42-CFL1 axis in human lung cancer cells
Source: Cell Commun Signal. 2021 Jul 28;19:80. doi: 10.1186/s12964-021-00763-6 (PMC8320060; doi:10.1186/s12964-021-00763-6)
Supplement: Supplementary file 3 — Additional file 2. Figure S2. Efficient Hhex knockdown and overexpression were confirmed by Western Blot analysis, related to Figure 1. A549, H1792 cells were transfected with control (CTRL) or HHEX siRNA, pcDNA3.1 or pcDNA3.1-HHEX for 24h and then subjected to western blot analysis. The images are representative of three independent experiments with similar results. [file 12964_2021_763_MOESM3_ESM.pptx]

## Slide 1
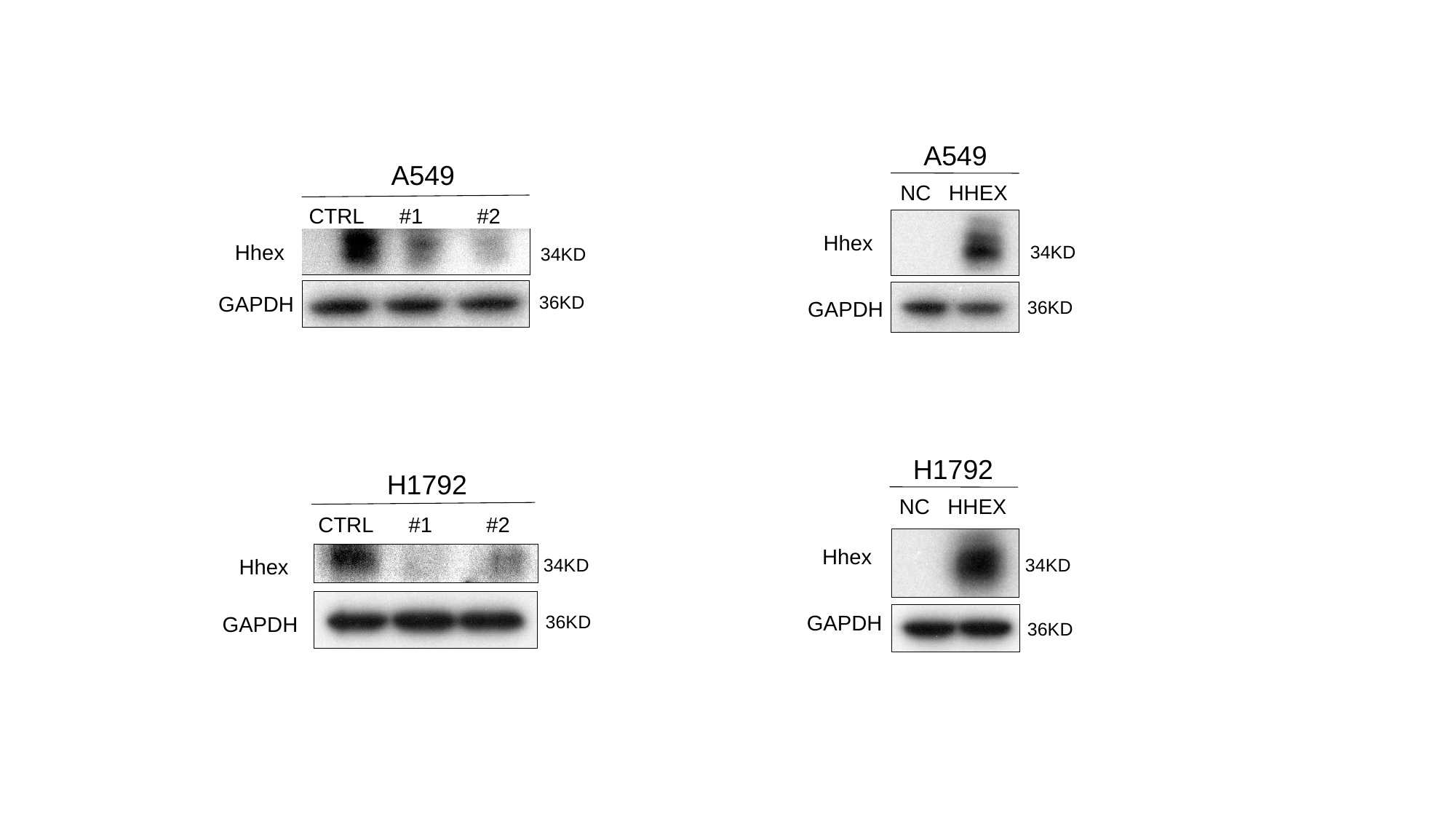

A549
A549
NC HHEX
CTRL #1 #2
Hhex
Hhex
34KD
34KD
GAPDH
36KD
GAPDH
36KD
H1792
H1792
NC HHEX
CTRL #1 #2
Hhex
Hhex
34KD
34KD
GAPDH
36KD
GAPDH
36KD
