# Supplementary material for: Hhex inhibits cell migration via regulating RHOA/CDC42-CFL1 axis in human lung cancer cells
Source: Cell Commun Signal. 2021 Jul 28;19:80. doi: 10.1186/s12964-021-00763-6 (PMC8320060; doi:10.1186/s12964-021-00763-6)
Supplement: Supplementary file 6 — Additional file 5. Figure S5 RhoGDIA inhibited the Hhex-RhoA/CDC42 interaction minimally. (a) pcDNA3.1-HHEX-HA, pcDNA3.1-RHOGDIA-FLAG and pcDNA3.1-CDC42-MYC were co-transfected into HEK 293T cell. Cell lysate was immunoprecipitated with anti-HA antibody, HA, MYC, ACTB, FLAG were detected by western blot. The images are representative of three independent experiments with similar results. (b) pcDNA3.1-HHEX-HA, pcDNA3.1-RHOGDIA-FLAG and pcDNA3.1-RHOA-MYC were co-transfected into HEK 293T cell. Cell lysate was immunoprecipitated with anti-HA antibody, HA, MYC, ACTB, FLAG were detected by western blot. The images are representative of three independent experiments with similar results. [file 12964_2021_763_MOESM6_ESM.pptx]

## Slide 1
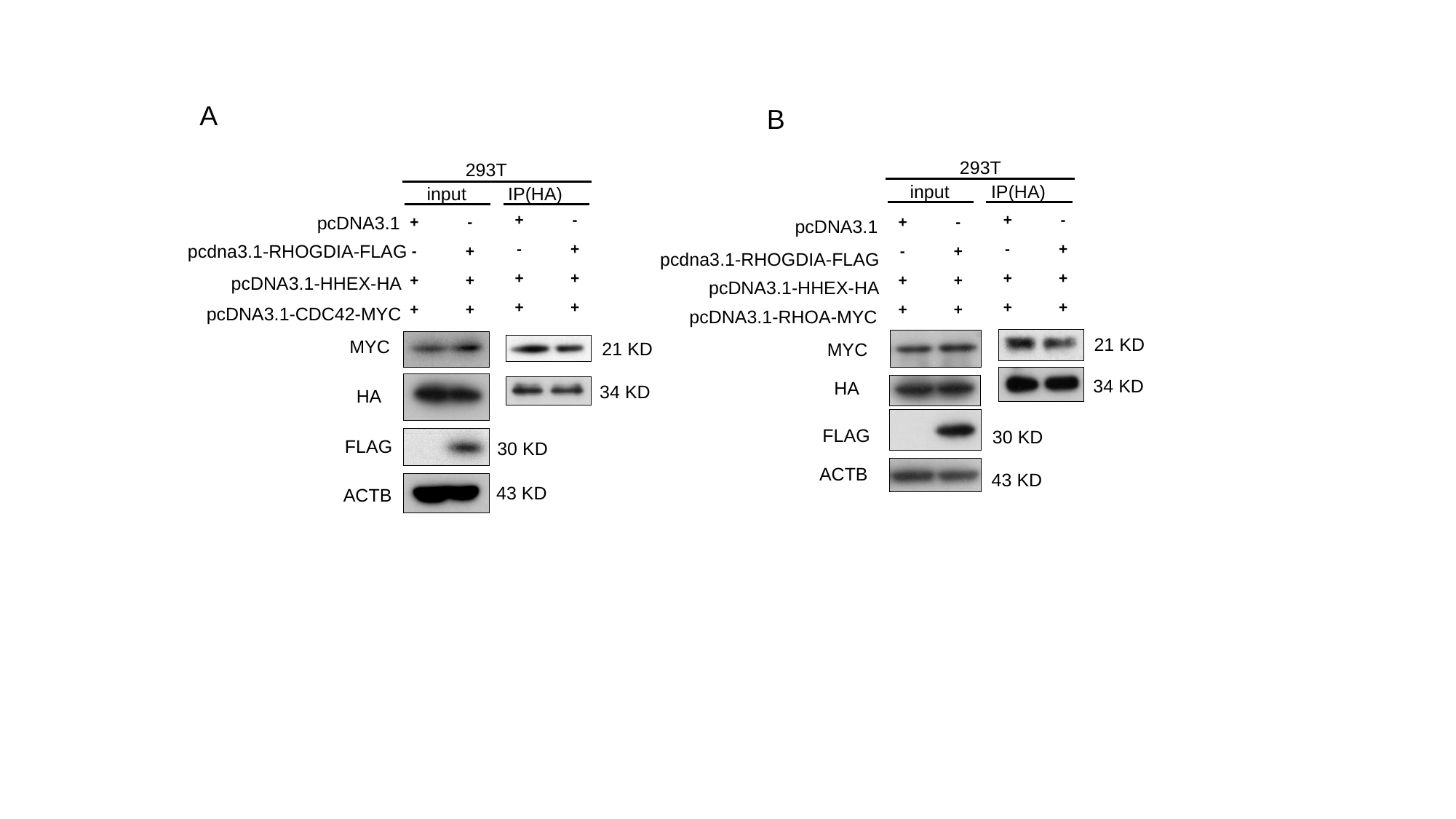

A
B
293T
293T
input
IP(HA)
input
IP(HA)
| + | - |
| --- | --- |
| - | + |
| + | + |
| + | + |
| + | - |
| --- | --- |
| - | + |
| + | + |
| + | + |
pcDNA3.1
| + | - |
| --- | --- |
| - | + |
| + | + |
| + | + |
| + | - |
| --- | --- |
| - | + |
| + | + |
| + | + |
pcDNA3.1
pcdna3.1-RHOGDIA-FLAG
pcdna3.1-RHOGDIA-FLAG
pcDNA3.1-HHEX-HA
pcDNA3.1-HHEX-HA
pcDNA3.1-CDC42-MYC
pcDNA3.1-RHOA-MYC
21 KD
MYC
21 KD
MYC
34 KD
HA
34 KD
HA
FLAG
30 KD
FLAG
30 KD
ACTB
43 KD
43 KD
ACTB
